# Parenting, Coparenting, and Adolescents’ Sense of Autonomy and Belonging After Divorce

**DOI:** 10.1007/s10964-024-01963-2

**Published:** 2024-03-30

**Authors:** Zoë Rejaän, Inge van der Valk, Wendy Schrama, Susan Branje

**Affiliations:** 1 Department of Youth and Family, Utrecht University, Utrecht, Netherlands; 2 Utrecht University School of Law, Molengraaff Institute for Private Law, Utrecht University, Utrecht, Netherlands

**Keywords:** Parental divorce, Adolescence, Coparenting, Parenting, Post-divorce living arrangements

## Abstract

Although there is ample evidence on the importance of experiencing autonomy and belonging for positive adolescent development and the supporting role of parents in this regard, most knowledge stems from intact families. As many youth grow up with divorced parents, this study tested longitudinal links between warm and autonomy supportive parenting and coparental cooperation and conflict on the one hand, and adolescents’ post-divorce autonomy and belonging on the other. Data consisted of three-wave self-report data of 191 Dutch adolescents (*M*_age_ = 14.36, 61.3% girls) and 227 divorced parents (*M*_age_ = 46.08, 74% mothers). Random-intercept cross-lagged panel models showed stable between-family differences, with autonomy relating positively to coparenting and parental autonomy support, and belongingness associating positively solely with parenting. No significant effects were found within families, meaning that changes in (co)parental behaviors did not predict adolescents’ experiences of autonomy and belonging or vice versa.

## Introduction

Parenting in post-divorce families is a complex task during adolescence, as adolescents learn to balance autonomy and belonging in relationships (Baumeister & Leary, 1995; Deci & Vansteenkiste, 2004), and coparenting relationships remain important (Beckmeyer et al., 2019). The current study focused on how parenting and coparenting affect adolescents’ functioning. Growing up in a post-divorce family has the potential to undermine adolescents’ experiences of belonging and autonomy (Friendly & Grolnick, 2009), which are crucial for their positive development in general (Ryan & Deci, 2000), but potentially even more, or more apparent, following a divorce. A major consequence of a divorce is the change from daily contact with family members to organized contact via post-divorce living arrangements, which also affects how routines of school, hobbies, and leisure time activities take place (Campo et al., 2012). As such, a lack of autonomy may become apparent through the feeling of having no say in decisions about living arrangements and related aspects in their daily living situation (Friendly & Grolnick, 2009). Another major consequence of parental divorce is that it usually puts pressure on the quality of parent-child and interparental relationships (Amato, 2010; Friendly & Grolnick, 2009), which may become apparent through a reduced sense of family belonging (Rejaän et al., 2021a). Thus far, studies have examined the role of parents in adolescents’ sense of autonomy and belonging, mostly in intact families. There is a lack of research addressing post-divorce experiences of autonomy and belonging, despite the assumption that each environment relates to the fulfillment of such needs in different ways and to a different extent (Ryan & Deci, 2000). Therefore, this study examined whether post-divorce parenting and coparenting are longitudinally associated with adolescents’ perceptions of autonomy in decision-making on post-divorce living arrangements and their sense of belonging to the family household(s) in which they reside.

### Growing up in Divorced Families

When parents separate or divorce, it often marks the beginning of a series of transitions in a child’s family environment, including future moves and changes in family composition (Amato, 2010). Each transition, however small or big, has direct consequences for their living situation, and with that, their daily lives. How families negotiate living arrangements at home and how youth’s interests are taken into account may thus be a recurring theme in divorced families with youth living at home. How such matters are dealt with is partially guided by (inter)national legislation. Dutch divorce law, for instance, obliges divorcing and separating parents with joint parental authority to formalize arrangements of care and upbringing of their minor children (De Bruijn, 2018). And, in accordance with the UN Convention on the Rights of the Child – which prescribes that children have the right to express their views on matters that affect them, and to have these views given due weight to by decision-makers – parents must state in a parenting plan how they have involved their minor children in the process of making living arrangements. Their involvement in these matters is thought to lead to more inclusive, more informed, and therefore better decision-making in the interests of children, as expressed by parents and children (Parkinson & Cashmore, 2008), as well as legal scholars (Mol, 2022; Parkinson & Cashmore, 2008; Schrama et al., 2021). However, parenting plans usually do not contain the option to renegotiate arrangements when the situation calls for it (De Bruijn, 2018), in accordance with their developmental needs (Berman, 2018; Kitterød & Lidén, 2020). To better understand how parents can best support youth’s development as they grow up in divorced families, this study focused on two key fundamental needs during adolescence: autonomy and belonging.

### Experiences of Autonomy and Belonging in Divorced Families

When growing up in a divorced family, adolescents’ need for autonomy may be reflected in the need to have a say in choices regarding their living situation (Deci & Ryan, 2008; Van Petegem et al., 2012). Imposed or unsatisfactory living arrangements may directly thwart adolescents’ sense of autonomous functioning. It thus seems warranted to give adolescents a voice in these living arrangements, and qualitative findings illustrate that this may take several forms (Birnbaum & Saini, 2012). The most obvious one is through actual participation, in which adolescents can express views, feel heard, and feel that these views matter (Mol, 2022). It seems that youth are generally interested in providing their input on living arrangements, whether they work, and how they can be improved (Birnbaum & Saini, 2012; Berman, 2018). Some youth, however, worry about having a decisional role in a situation with potentially conflicting loyalties and therefore choose not to participate (Cashmore & Parkinson, 2007; Campbell, 2008). Adolescents’ sense of autonomy may also be supported as long as they trust that their wishes and interests are sufficiently taken into account by others, for instance when children are younger (Berman, 2018), when they are unwilling or unable to choose (Cashmore, 2011; Haugen, 2010). Furthermore, perceptions of autonomy might be higher when there is flexibility of arrangements and freedom to deviate from them (Birnbaum & Saini, 2015). Some adolescents find scheduled contact unnecessarily rigid and restrictive (Kelly, 2007) or prefer the opportunity to spend additional time with a parent when they please (Kitterød & Lidén, 2020). Finally, given the different ways youth can and may prefer to be involved in decision-making processes, satisfaction with established arrangements could also be considered. Although some degree of influence seems to be connected to their satisfaction with the arrangements (Berman, 2018), having a say does not necessarily mean that youth are happier with their living arrangements. However, being unhappy about the living arrangements may reinforce the need for control, and exacerbate the experience of low autonomy in decision-making (Cashmore, 2011). In sum, adolescents’ autonomy when growing up in divorced families may depend on whether they have a say in their living arrangements, whether they feel like their opinions matter, and whether they experience flexibility in and satisfaction with arrangements.

Another key factor in adolescents’ development is experiencing a sense of belonging: feeling connected to parents and having a secure base from which they can explore the world (Ryan & Deci, 2000; Griffin et al., 2017). Compared to their peers from intact families, adolescents from divorced families are prone to experience a weaker sense of belonging to their families (King et al., 2018; Rejaän et al., 2021a). Within post-divorce families, research has also demonstrated differences in the extent to which adolescents experience a sense of belonging to maternal and paternal households, which could partially be explained by the generally unequal amount of time that adolescents typically spend with their mothers versus their fathers (Rejaän et al., 2021a). Experiencing autonomy in post-divorce decision-making about living arrangements may help adolescents navigate and maximize their desired time in both households, and thereby may support their sense of family belonging (Birnbaum & Saini, 2012; Lodge & Alexander, 2010). Vice versa, experiencing belonging may empower adolescents to become proactive in managing their need for autonomy in decision-making (i.e., *need crafting*; Laporte et al., 2021).

### The Role of Parents in Adolescents’ Post-Divorce Autonomy and Belonging

Adolescents’ experiences of autonomy and belonging are dependent on their families’ support (Neale & Flowerdew, 2007), and a broad perspective on the divorced family system is necessary to understand these experiences (Cox & Paley, 2003). Particularly the parent-adolescent subsystems and the coparental subsystem are important in this context. Within the parent-adolescent subsystems, parents’ child-directed warmth and autonomy support appear especially relevant (Grolnick et al., 1997; Joussemet et al., 2008). When it comes to actively involving youth in decisions on living arrangements, parents ideally engage in warm and sensitive interactions while acknowledging their children’s perspective, providing choice, and encouraging exploration (Vansteenkiste & Soenens, 2015; Fousiani et al., 2014). Rather than prioritizing their own interests, autonomy supportive parents allow or tolerate differences of opinion and ideas, and encourage children regardless (Soenens et al., 2017). This type of parenting by fathers and mothers has been shown to help adolescents in learning to act upon their own interests (Assor, 2012; Fousiani et al., 2014), and presumably applies to youth in divorce situations too. Furthermore, warm and autonomy supportive parenting helps facilitate family belongingness (King et al., 2018).

Adolescents’ autonomy in decision-making and belonging to both households likely also depends on the coparental relationship (Cox & Paley, 2003; Markham et al., 2017). In the (re)negotiation of living arrangements, parents are known to often make post-divorce decisions based on their own logistical, financial, and relational considerations, and interests of parents may collide (Holt, 2016; Russell et al., 2016). In the post-divorce coparenting literature, a distinction is usually made between positive coparenting dimensions or behaviors, such as cooperation, and negative dimensions, such as conflict (e.g., Beckmeyer et al., 2019; Rejaän et al., 2021b). Cooperative parents engage in frequent communication and collaboration, and a low level of conflict is most likely to create a supportive environment that promotes both their own and their children’s positive adjustment after divorce (Sigal et al., 2011). Not all parents are able to achieve a cooperative relationship after divorce, as for some, conflicts continue to dominate the relationship (Beckmeyer et al., 2019; Rejaän et al., 2021b). In line with qualitative findings (Berman, 2018), coparental hostility and uncooperativeness were expected to serve as barriers to adolescents’ autonomy and belonging, while lack of conflict and coparenting cooperativeness are more likely to support it.

To better understand the role of parents in adolescents’ experiences of autonomy and belonging in divorced families, there are some important considerations. Firstly, since growing up in post-divorce families is often characterized by (need for) changes in living arrangements shortly and long after parents separate (Berman, 2018; Kitterød & Lidén, 2020), families with varying levels of time since the divorce are relevant to study. Secondly, the role of adolescents’ age and the amount of time should not be ignored in this matter, because youth generally develop better decision-making skills and (desire to) gain more participatory rights within their families as they get older (Berman, 2018; Palmer et al., 2017) and when more time since the divorce has passed (Neale & Flowerdew, 2007). The same goes for whether parents were married before their divorce or not, as the former group is much more likely to make formal living arrangements than the latter (De Bruijn, 2018). Thirdly and finally, there is a need to use methods that can investigate the order of effects and can distinguish between-family associations from within-family effects, such as random-intercept cross-lagged panel models (Hamaker et al., 2015).

## Current Study

While the importance of experiencing autonomy and belonging for positive adolescent development and the supporting role of parents in this regard is well established, most of this knowledge stems from intact families. The current study tested whether post-divorce parenting and coparenting behaviors were associated with divorce-specific experiences of autonomy and belonging, both across and within families. Specifically, it focused on the extent to which adolescents experienced autonomy in decision-making about living arrangements and their perceptions of belonging to the post-divorce households in which they reside. In line with a self-determination perspective and prior empirical evidence, it was hypothesized that adolescents whose parents reported higher levels of warm and autonomy supportive parenting, and more cooperative and harmonious coparenting, would report higher levels of autonomy and belonging themselves. Autonomy and belonging were also expected to be positively associated. Additionally, changes in parent-reported parenting and coparenting were hypothesized to be related to changes in adolescents’ sense of autonomy and belonging over time.

## Methods

### Procedure

This study used multi-informant self-report data of adolescents and parents from divorced families that were gathered between 2019 and 2022 within the research project “Where do I belong?”, a three-wave study with 9-month intervals. To optimize sample size and heterogeneity, divorced families throughout the Netherlands were recruited in a variety of ways: Through schools, legal and health care professionals, targeted (online) advertisements, and snowball sampling. After receiving detailed information about the study, parents and adolescents each signed online informed consent forms. Respondents individually filled out online questionnaires, taking 40 to 60 minutes, and received a small monetary compensation for their contribution per measurement wave. Families could withdraw their participation at any time during or after participation, without stating reasons. The research protocol was approved by the Faculty Ethics Review Board of the Social Sciences Faculty of Utrecht University (Protocol code: FETC18-008).

### Sample

The study sample consisted of participants from 146 different families: 191 adolescents (61.3% girls), and 227 parents (74% mothers). Adolescents’ age ranged from 11 to 18 years old at T1 (*M* = 14.36, *SD* = 1.89). The majority (87.8%) attended secondary school: 25% of the adolescents followed the pre-vocational education track, 29.2% the pre-professional track, and 33.5% the pre-academic track. The remaining respondents attended the final year of primary education (3.7%), higher education (7.5%) or no education (1.1%). With regard to their cultural background, 97.4% identified as (partly) Dutch, and most considered themselves non-religious (77.1%) or Christian (16.5%). As for parents, the majority was born in the Netherlands (92.1%), and considered themselves non-religious (60.7%), Christian (32.6%), or other (6.7%). Their age ranged from 34 to 59 years old at T1 (*M* = 46.08, *SD* = 5.06), and highest attained education varied from primary or secondary education (9.6%) to vocational (20.2%), professional (33.1%), or academic education (37.1%), and most parents had a paid job at T1 (92.1%),

Families participated with either one (*N* = 105 families), two (*N* = 37) or three adolescent children (*N* = 4), and with either both parents (*N* = 40) or one parent. The majority of parents used to be married or were in a registered partnership (72.6%), whereas the others were never married. On average, the children were 7.54 years old (*SD* = 4.06) during the parental divorce or separation, and the time since the divorce during the first wave ranged from 0 to 16 years (*M* = 6.83, *SD* = 4.06). Adolescents’ post-divorce living arrangements were measured with the Residential Calendar (Sodermans et al., 2014), which showed that, at T1, adolescents on average spent *M* = 2.33 (*SD* = 1.63) days and nights per week in their fathers’ homes, and the remaining time in their mothers’ homes. Based on the time adolescents spent in their parental homes, their living arrangements can be categorized as follows: 21.1% of the adolescents lived solely [100% of the time] with their mother, 27.6% lived mostly [67–99%] with their mother, 46.5% lived a roughly equal amount of time with both parents [33–66%], and some lived mostly (2.7%) or solely (2.2%) with their father.

Out of the initial study participants, 145 adolescents (75.9%), 50 fathers (84.7%) and 132 mothers (78.6%) still participated in the final measurement wave. Adolescents who participated in all three waves reported significantly higher levels of maternal family belonging at T1 (*M* = 4.33, *SD* = 0.58) than adolescents who did not (*M* = 4.06, *SD* = 0.63), *t*(176) = −2.52, *p* = 0.013. Furthermore, mothers who participated in all three waves reported significantly more autonomy support at T1 (*M* = 3.75, *SD* = 0.34) than mothers who did not (*M* = 3.54, *SD* = 0.58). No other differences in background or study variables were found.

As the current study focused on autonomy in decision-making about living arrangements, adolescents were asked whether they currently had arrangements regarding when they resided with their mothers and fathers. At T1, *N* = 155 adolescents (81.2%) reported having established living arrangements. Adolescents with arrangements had significantly more cooperative coparents (*M* = 3.63, *SD* = 0.93 versus *M* = 2.90, *SD* = 1.28), and on average spent more time in their father’s home (i.e., less in their mother’s home) on a weekly basis than their peers without established living arrangements (*M* = 2.57, *SD* = 1.54 versus *M* = 0.87, *SD* = 1.36).

### Measures

#### Parent-reported warmth and autonomy support

Parental warmth towards their children was measured with the Warmth subscale of the Coparenting Behavior Questionnaire (CBQ; Schum & Stolberg, 2007). Mothers and fathers assessed 7 items on a Likert scale (1 = *almost never*, 5 = *almost always*), such as “I show my child that I care about them”, and “I enjoy spending time with my child”. Reliability of both maternal and paternal measures were considered good as, respectively, they ranged from α = 0.86 to 0.88, and from α = 0.84 to 0.91 across waves.

Parental autonomy support was measured in terms of their tolerance to differences of opinions and ideas towards their children, by using the Balanced Relatedness scale (Shulman et al., 1997). Mothers and fathers assessed 7 items on a Likert scale (1 = *strongly disagree*, 4 = *strongly agree*), such as “I give my child space to have their own ideas”, and “I take my child’s opinion into account”. Reliability of maternal measures was good (0.82 ≤ α ≥ 0.87) and reliability of paternal measures was acceptable to good (0.64 ≤ α ≥ 0.80). Per parenting dimension, items were combined into average scores, with higher scores indicating higher levels of warmth and more autonomy support.

With only 50 fathers versus 132 mothers participating in the study, the aim was to make maximum use of all data. This means that in families with only one participating parent, those parents’ scores were used, whereas in families with two participating parents, combined scores of fathers’ and mothers’ parenting were used. Across waves, correlations between maternal and paternal warmth were small and (mostly) non-significant (−0.15 ≤ *r* ≥ 0.04), as were correlations between maternal and paternal autonomy support (−0.17 ≤ *r* ≥ 0.39). To account for potential differences in adolescents’ *exposure* to each parents’ parenting due to the amount of time they stay with each parent, scores of parental warmth and autonomy support were weighted using the categorized living arrangements (see Sample section). This means that weighted scores were composed of either 0%, 25%, 50%, 75% or 100% of mother- and father-reported parenting. For example, only mothers’ scores were used for adolescents who indicated living solely with their mothers, whereas mothers’ and fathers’ scores were averaged for those living a roughly equal amount of time with both their parents. Paired samples *t*-tests showed no significant differences between fathers’ and mothers’ reports of warmth or autonomy support, indicating that their scores could be combined.

#### Parent-reported coparental cooperation and conflict

Coparenting dimensions were measured with parents’ reports on the Coparenting Behavior Questionnaire (Schum & Stolberg, 2007), with items on a Likert scale (1 = *almost never*, 5 = *almost always*). Coparental respect and cooperation towards their coparent was measured with the Coparental Respect/Cooperation subscale, consisting of 8 items such as “I want my child to have a good relationship with my ex-partner”, and “My ex-partner helps out when I need to change my child’s schedule”. Reliability was good for mother-reported cooperation (0.92 ≤ α ≥ 0.95) as well as father-reported cooperation (0.83 ≤ α ≥ 0.91).

Parents’ perceived conflict with and hostility towards the other parent was measured with the Coparental Conflict subscale, consisting of 10 items, such as “Me and my ex-partner get angry when we talk to each other”, and “Me and my ex-partner argue in front of our child(ren)”. Conflict measures were also reliable across waves: mother-reported conflict (0.75 ≤ α ≥ 0.89) and father reported-conflict (0.89 ≤ α ≥ 0.90).

Per coparenting dimension, items in each scale were combined into average scores, with higher scores indicating higher cooperation and higher conflict. Again, the aim was to utilize all available parent-report data, using a single score in families with one participating parent, and a combined score in families with two participating parents. Because correlations between mothers’ and fathers’ reports were high for both cooperation (0.39 ≤ *r* ≥ *0*.49) and conflict (0.83 ≤ *r* ≥ 0.85), and paired samples *t*-tests showed no significant mean differences in maternal versus paternal reports, their individual scores were averaged into single scores.

#### Adolescents’ sense of autonomy in decision-making

Adolescents answered questions regarding their living arrangements in each wave. At Wave 1, they were asked to indicate whether arrangements had been made after the divorce (*Yes/No*), and 155 adolescents indicated *Yes*. At following waves, they were asked whether their living arrangements had changed in the last 9 months (*Yes/No*), to which 110 adolescents indicated *Yes* at Wave 2, and 103 adolescents indicated *Yes* at Wave 3. In each wave, those who indicated Yes were asked to report on four items assessing their sense of autonomy in this regard: a) Their participation in the decision-making process: “Did you participate in conversations about when you will reside with your father or mother?” (1 = *no, just my parents*, 2 = yes, *my parents and I decided together*, 3 = *yes, it was my decision*); b) Their perception on the importance of their opinion: “How important is your opinion about living arrangements to your parents?” (1 = *not at all important*, 5 = *very important*); c) Flexibility in arrangements: “Is there room to deviate from established living arrangements in consultation?” (1 = *not at all*, 5 = *very much*); and d) Their satisfaction with the arrangements: “Are you satisfied with the current living arrangements?” (1 = *not at all*, 5 = *very much*). To investigate the underlying structure of these four items, data were analyzed by means of principal component analysis. In each wave, one component with an eigenvalue above 1.0 was identified, explaining between 45% and 57% of the total variance. This indicates that these items can be thought of as representing one underlying factor, which in this study was label as adolescents’ autonomy in decision-making on living arrangements. As such, items were combined into a mean score, with higher values indicating a higher sense of autonomy.

#### Adolescents’ sense of family belonging

Adolescents’ perceptions of family belonging were measured as a mean score of four items, each with five response options (1 = *very little*, 5 = *very much*), adapted from King and Boyd (2016), see Rejaän et al. (2021a). Adolescents reported separately on their sense of belonging to each post-divorce household: “How much do you feel your family in your father’s/mother’s home understands you?”, “How much do you feel you and your family in your father’s/mother’s home have fun together?”, “To what extent do you feel your family in your father’s/mother’s home pays attention to you?”, and “How much do you feel you want to leave your father’s/mother’s home? (reversely coded). Reliability of family belonging measures were considered good across waves, with α ranging from 0.74 to 0.77 for maternal belonging, and a consistent α = 0.87 for paternal belonging. Similar to the parenting measures in this study, and family belonging measures of Rejaän et al. (2021), a single, weighted score of adolescents’ family belonging was used.

### Strategy of Analyses

Mplus Version 8.1 (Muthén & Muthén, 1998–2018) was used to run four random intercept cross-lagged panel models (RI-CLPM; Hamaker et al., 2015), assessing associations between(co)parenting, adolescent autonomy in decision-making, and adolescent family belonging for each of the four (co)parenting dimensions separately. All variables had missing data (mostly due to attrition), which – except for adolescent autonomy (see measures) – ranged from 1.3% to 6.8% for T1 variables, from 15.7% to 20.4% for T2 variables, and from 21.5% to 29.3% for T3 variables. Although Little’s missing completely at random test (1988) was significant, the normed chi-square of 1.20 was acceptable (i.e., χ²/df <2; Bollen, 1989), which implied a small violation of the missing completely at random assumption that could be handled by means of Full Information Maximum Likelihood estimation. Furthermore, since multiple adolescents from the same family were included in the study, which violates the assumption of independence of the data, within-family dependency (i.e., the fact that data are nested) was accounted for by using the ‘complex’ sample cluster feature in combination with the Maximum Likelihood Robust (MLR) estimator (Muthén & Muthén, 1998–2018).

For each (co)parenting dimension, a separate RI-CLPM was specified. Each RI-CLPM was tested in two steps. First, a fully unconstrained model was tested, and secondly, a model with time-constraints to stability pathways, cross-lagged pathways, and within-wave correlations. Model results were controlled for adolescent age, the amount of time since the divorce or separation, and whether or not parents were formerly married (dummy coded). These covariates were included in the model by regressing the observed scores of (co)parenting dimensions and adolescent needs on the covariates. Overall model fit was evaluated with the comparative fit index and Tucker-Lewis Index (CFI and TLI >0.90 = acceptable; >0.95 = good fit), the root-mean-square error of approximation and the standardized root-mean-square-residual (RMSEA and SRMR <0.08 = acceptable; <0.06 = good fit). To test time-equality constraints, the Akaike and adjusted Bayesian Information Criteria were compared (lower AIC and aBIC values represent better models), and chi-square difference tests were computed. If the constraints did not worsen fit, they were retained in the model for reasons of parsimony.

## Results

Table 1 presents the descriptive statistics and bidirectional correlations between all study variables included in the random intercept cross-lagged panel models. As described in the Methods section, scores on parental warmth and autonomy support reflect weighted scores of fathers and mothers, and scores on parental cooperation and conflict reflect averaged scores of fathers and mothers.

**Table 1:** Correlations between and descriptive statistics of all study variables

	1	2	3	4	5	6	7	8	9	10	11
Time 1											
1. Age ^A^											
2. Time s/ divorce ^A^	0.23**										
3. Divorce type ^A^	−0.08	0.02									
4. Warmth ^P^	−0.23**	0.08	−0.09								
5. Autonomy support ^P^	0.00	0.13	−0.02	0.30**							
6. Cooperation ^C^	−0.08	−0.05	0.05	0.03	0.13						
7. Conflict ^C^	−0.02	−0.17*	−0.02	−0.08	−0.11	−0.58**					
8. Autonomy ^A^	0.12	−0.03	0.02	0.04	0.25**	0.30**	−0.30**				
9. Belonging ^A^	−0.15*	−0.05	−0.03	0.26**	0.10	−0.06	−0.06	0.52**			
Time 2											
10. Warmth ^P^	−0.11	0.17*	−0.15	0.68**	0.37**	0.05	−0.08	0.01	0.22**		
11. Autonomy support ^P^	0.18*	0.17*	−0.05	0.14	0.38**	0.01	−0.16*	0.00	0.01	0.28**	
12. Cooperation ^C^	−0.15	−0.09	0.04	0.02	0.05	0.91**	−0.56**	0.32**	0.10	0.04	−0.02
13. Conflict ^C^	−0.01	−0.17*	−0.01	−0.07	−0.16	−0.47**	0.76**	−0.27**	−0.07	−0.17*	−0.29**
14. Autonomy ^A^	0.06	0.06	−0.11	−0.04	0.09	0.28**	−0.28**	0.45**	0.43**	0.24*	0.11
15. Belonging ^A^	−0.22**	0.08	−0.09	0.27**	0.07	0.11	−0.16	0.30**	0.63**	0.36**	0.07
Time 3											
16. Warmth ^P^	−0.08	0.21*	−0.08	0.63**	0.31**	−0.06	−0.04	−0.08	0.21*	0.69**	0.22**
16. Autonomy support ^P^	0.07	0.16	−0.08	0.16	0.42**	−0.05	−0.10	0.12	0.17	0.12	0.25**
18. Cooperation ^C^	−0.09	0.03	0.00	0.04	0.12	0.86**	−0.59**	0.32**	0.03	0.02	0.08
19. Conflict ^C^	0.01	−0.17*	0.03	−0.12	−0.17*	−0.54**	0.63**	−0.36**	−0.06	−0.16*	−0.16
20. Autonomy ^A^	0.18	0.09	−0.21*	0.04	0.20	0.21*	−0.24*	0.55**	0.35**	0.09	0.09
21. Belonging ^A^	−0.18*	0.07	−0.06	0.23**	0.08	0.14	−0.14	0.39**	0.59**	0.26**	−0.04
*N*	188	188	187	178	178	185	185	155	178	160	160
*M*	14.36	6.83	0.27	4.50	3.69	3.52	1.90	3.47	4.21	4.39	3.73
*SD*	1.89	4.06	0.44	0.45	0.35	1.03	0.74	0.58	0.54	0.56	0.38
		12	13	14	15	16	17	18	19	20	21
Time 2											
13. Conflict ^C^		−0.47**									
14. Autonomy ^A^		0.32**	−0.21*								
15. Belonging ^A^		0.12	−0.14	0.55**							
Time 3											
16. Warmth ^P^		−0.02	−0.15	0.04	0.24**						
17. Auto. Support ^P^		−0.07	−0.22**	0.10	0.06	0.30**					
18. Cooperation ^C^		0.86**	−0.51**	0.15	0.06	−0.03	0.03				
19. Conflict ^C^		−0.53**	0.66**	−0.18	−0.14	−0.05	−0.19*	−0.55**			
20. Autonomy ^A^		0.25*	−0.20*	0.64**	0.40**	0.00	0.30**	0.26**	−0.34**		
21. Belonging ^A^		0.20*	−0.14	0.41**	0.66**	0.26**	0.18*	0.19*	−0.23**	0.50**	
*N*		161	161	110	154	142	142	150	150	103	136
*M*		3.45	1.79	3.57	4.10	4.37	3.72	3.33	1.72	3.58	4.01
*SD*		1.17	0.67	0.57	0.62	0.51	0.36	1.25	0.56	0.66	0.61

The first three variables are covariates in RI-CLPMs: 1) adolescents’ age in years, 2) the amount of time since the parental divorce in years, and 3) divorce type, i.e., whether parents were previously married (dummy coded, 0 = previously married, 1 = previously not married). Superscripts: ^A^ = adolescent, ^P^ = parental, ^C^ = coparental. Parental scores reflect weighted or averaged scores of fathers and mothers, as reported in the Method section

*p ≤ 0.05; **p ≤ 0.001

Table 2 shows the fit indices of the specified random intercept cross-lagged panel models for each (co)parenting dimension. For the coparenting models, the analytical plan could not be executed as intended, as most variance in cooperation and conflict was explained by the random-intercept, and variance in the within-person centered variables was small and not consistently significant. Alternatively, constrained and unconstrained RI-CLPMs were specified in which coparenting dimensions were solely included at the between-family level. Specifically, RIs were created for all variables, the measurement error variances of the observed coparenting variables were kept unconstrained, and within-person centered variables were created only for adolescents’ sense of autonomy and belonging.

**Table 2:** Model fit indices for all RI-CLPMs

	χ²	df	CFI	TLI	RMSEA	SRMR	AIC	aBIC	*p* value χ² diff. test
Warmth									
1. Unconstrained	1.397	3	1.000	1.064	0.000	0.012	1616.88	1621.84	0.411
2. Constrained	14.257	15	1.000	1.006	0.000	0.086	1605.00	1609.20	
Autonomy Support									
1. Unconstrained	9.481	3	0.982	0.622	0.107	0.021	1541.50	1546.47	0.655
2. Constrained	16.377	15	0.996	0.984	0.022	0.073	1527.61	1531.81	
Cooperation ^a^									
1. Unconstrained	25.071	19	0.991	0.971	0.041	0.038	2199.69	2203.64	0.310
2. Constrained	31.025	24	0.990	0.973	0.040	0.058	2196.05	2199.68	
Conflict ^a^									
1. Unconstrained	34.881	19	0.966	0.889	0.067	0.073	1962.83	1966.78	0.267
2. Constrained	41.682	24	0.963	0.902	0.063	0.081	1959.38	1963.01	

^a^Adjusted RI-CLPMs with estimates for coparenting variables only at the between-level

All RI-CLPMs have an acceptable fit. The difference in AIC/aBIC between the constrained and unconstrained models is larger than 10 in the parenting models, and larger than 2 in the coparenting models, which together with non-significant chi-square difference tests indicates evidence in favor of the constrained models (Satorra & Bentler, 2001). The final models for parental warmth and autonomy support are reported in Table 3, which contains four types of effects: between-person correlations, within-person stability effects, within-person cross-lagged effects, and within time associations (within-person correlated change). The final models for coparental cooperation and conflict are presented in Table 4, which due to the model adjustments contains between-person correlations for all variables, and within-person associations and effects solely for adolescent outcomes.

**Table 3:** Parameter estimates for the constrained RI-CLPMs modeling parenting with adolescent autonomy and belonging

	Parental warmth				Parental autonomy support				
	*B*	*SE B*	*p*	*β*	*B*	*SE B*	*p*	*β*	
Between-person correlations									
Parenting ←→ Autonomy	0.03	0.02	0.162	0.16	**0.04**	**0.01**	**0.004**	**0.35**	
Parenting ←→ Belonging	**0.05**	**0.02**	**0.004**	**0.31**	**0.03**	**0.01**	**0.005**	**0.32**	
Autonomy ←→ Belonging	**0.13**	**0.02**	**0.000**	**0.68**	**0.14**	**0.03**	**0.000**	**0.74**	
Within-person Correlations T1									
Parenting ←→ Autonomy	0.02	0.01	0.052	**0.22**	0.03	0.01	0.134	0.25	
Parenting ←→ Belonging	0.01	0.01	0.492	0.08	−0.01	0.01	0.244	−0.17	
Autonomy ←→ Belonging	**0.04**	**0.02**	**0.004**	**0.43**	**0.05**	**0.02**	**0.008**	**0.38**	
Within-person Cross-lagged effects^a^				T1-2 T2-3				T1-2	T2-3
Parenting → Autonomy	−0.30	0.19	0.114	−0.27 −0.15	−0.01	0.16	0.520	−0.01	−0.01
Parenting → Belonging	0.22	0.16	0.178	0.14 0.12	−0.14	0.13	0.275	−0.08	−0.11
Autonomy → Parenting	0.05	0.07	0.502	0.09 0.04	0.01	0.07	0.899	0.07	0.06
Autonomy → Belonging	−0.16	0.12	0.182	−0.16 −0.12	−0.14	0.13	0.274	−0.47	−0.11
Belonging → Parenting	0.00	0.10	0.980	0.00 0.00	−0.09	0.07	0.192	−0.14	−0.21
Belonging → Autonomy	0.28	0.15	0.057	0.32 0.26	0.17	0.17	0.382	0.26	0.23
Within-person Stability paths^a^				T1-2 T2-3				T1-2	T2-3
Parenting	−0.09	0.24	0.699	−0.11 −0.07	−0.16	0.13	0.233	−0.11	−0.15
Autonomy	**−0.37**	**0.15**	**0.016**	**−0.50 −0.24**	−0.23	0.23	0.362	−0.47	**−0.22**
Belonging	0.22	0.19	0.231	0.18 0.22	0.28	0.16	0.084	0.25	0.35
Within-person Correlated change T2 & T3				T2 T3				T2	T3
Parenting ←→Autonomy	0.00	0.01	0.806	0.06 0.02	0.00	0.01	0.828	0.03	0.03
Parenting ←→ Belonging	**0.03**	**0.01**	**0.046**	**0.32 0.23**	0.01	0.01	0.586	0.05	0.06
Autonomy ←→ Belonging	**0.04**	**0.02**	**0.028**	**0.44 0.25**	0.03	0.02	0.100	0.27	0.21

Significant estimates are in boldface. Model results are controlled for adolescent age, time since the divorce, and whether or not parents were formerly married (dummy coded, 0 = previously married, 1 = never married)

^a^T1-2 and T2-3 paths are constrained to equality

**Table 4:** Parameter estimates for the adjusted constrained RI-CLPMs modeling coparenting with adolescent autonomy and belonging

	Coparental cooperation				Coparental conflict				
	*B*	*SE B*	*p*	*β*	*B*	*SE B*	*p*	*β*	
Between-person correlations									
Coparenting ←→ Autonomy	**0.17**	**0.05**	**0.000**	**0.37**	**−0.09**	**0.03**	**0.000**	**−0.41**	
Coparenting ←→ Belonging	0.04	0.04	0.348	0.09	−0.03	0.02	0.270	−0.11	
Autonomy ←→ Belonging	**0.13**	**0.02**	**0.000**	**0.69**	**0.14**	**0.03**	**0.000**	**0.73**	
Correlations T1									
Autonomy ←→ Belonging	**0.05**	**0.02**	**0.008**	**0.39**	**0.05**	**0.02**	**0.007**	**0.39**	
Cross-lagged effects^a^				T1-2 T2-3				T1-2	T2-3
Autonomy → Belonging	−0.15	0.12	0.199	−0.14 −0.12	−0.18	0.12	0.112	−0.16	−0.14
Belonging → Autonomy	0.22	0.18	0.207	0.25 0.22	0.22	0.19	0.250	0.17	0.16
Stability paths ^a^				T1-2 T2-3				T1-2	T2-3
Autonomy	−0.33	0.22	0.124	−0.42 −0.23	−0.25	0.27	0.361	−0.30	−0.19
Belonging	0.27	0.16	0.096	0.22 0.29	0.27	0.17	0.098	0.22	0.29
Correlated change				T2 T3				T2	T3
Autonomy ←→ Belonging	**0.04**	**0.02**	**0.038**	0.30 0.21	0.03	0.02	0.127	0.25	0.19

Significant estimates are in boldface. Model results are controlled for adolescent age, time since the divorce, and whether or not parents were formerly married (dummy coded, 0 = previously married, 1 = never married)

^a^T1-2 and T2-3 paths are constrained to equality

### (Co)Parenting, Adolescent Autonomy, and Family Belonging

Regarding our hypotheses that warm and autonomy supportive parenting and cooperative and harmonious coparenting would be associated with adolescents’ post-divorce sense of autonomy and belonging, several statistics in Tables 3 and 4 should be considered.

#### Between-family level

Associations between the random intercept variables provide information about how stable between-family differences in one variable are associated with those in another variable. All models show such significant positive associations. Specifically, they show that adolescents whose parents reported relatively more warmth tended to report a stronger sense of family belonging compared to their peers, but there is no significant association between parental warmth and adolescent autonomy. Furthermore, adolescents whose parents reported relatively more autonomy support, also reported more autonomy in decision-making and family belonging than their peers.

Both coparenting models show significant associations among coparenting dimensions and autonomy, but not among coparenting and belonging, indicating that divorced parents who were more cooperative and had less conflict with their coparent have children who generally reported more autonomy compared to their peers.

Taken together, these results only partially support our hypotheses: Across families, adolescents’ autonomy is indeed associated with parental autonomy support, coparental cooperation and coparental conflict, but not with parental warmth. Furthermore, adolescents’ belonging is indeed associated with parental warmth and autonomy support, yet not with coparental cooperation or conflict. All four models show that adolescents who reported more autonomy compared to other adolescents tended to also report a stronger sense of belonging.

#### Within-family level

Within-family statistics shed light on whether changes in (co)parenting predict changes in adolescents’ autonomy and belonging over time. As Tables 3 and 4 shows, there are some significant within-family correlated change estimates. Across all models, adolescent autonomy and belonging are concurrently related at T1, but only in the parental warmth and coparental cooperation model are these variables significantly correlated at subsequent waves. This significant association suggests that at times when adolescents experienced more autonomy in living arrangement decisions, they also experienced higher belonging. Additionally, the parental warmth model shows correlated change between parental warmth and adolescent belonging, suggesting that belonging was higher at time points when parental warmth was higher. No other significant correlated changes are found.

With regard to estimates for within-family cross-lagged effects, there are no significant estimates, thereby rejecting our hypotheses. Namely, when parents displayed relatively more warmth, autonomy support, cooperation, or conflict than they usually did, this had no significant effect on adolescents’ subsequent autonomy or belonging. Fluctuations in adolescents’ experiences of autonomy also did not have a significant effect on subsequent belonging or vice versa.

#### Covariates

All models include several covariates to control for their effects on adolescents’ autonomy and belonging. Parents of younger adolescents on average reported more parental warmth at Wave 1 and 2, and more coparental cooperation across waves. Being older predicted more perceived autonomy in living arrangements at Wave 1, and a weaker sense of family belonging across waves. The amount of time since the parental divorce or separation positively and significantly predicted parental warmth at Wave 2 and 3, and negatively predicted parent-reported conflicts at Wave 1, in the way that the more time had passed since the divorce, the less coparental conflicts parents reported. Lastly, there is a significant effect of the type of divorce on adolescents’ autonomy at Wave 3, indicating that adolescents whose parents were previously married reported more autonomy in living arrangements during the final wave than adolescents whose parents were never married. Results of the final models are the same when adolescent age, time since the divorce, and type of divorce are not controlled for.

#### Sensitivity analyses

Due to the small and (mostly) non-significant correlations between paternal and maternal reports on warmth and autonomy support, sensitivity analyses were performed to examine whether the use of combined, weighted scores of parents’ self-reported parenting affected the results of the RI-CPLMs. The models were specified using only mothers’ self-reported parenting. The results of these sensitivity analyses were comparable with the results reported in Table 3: On both the between-family and within-family level, significant associations remained similar in direction and strength, while non-significant remained non-significant.

## Discussion

Although there is ample evidence on the importance of experiencing autonomy and belonging for positive adolescent development and the supporting role of parents in this regard, most knowledge stems from intact families. Empirical research on these associations in non-intact families is much needed. A substantial amount of youth will face a parental divorce before they reach young adulthood, potentially interfering with key developmental tasks in adolescence. One of the most direct consequences of a divorce is the change from self-evident contact with both parents to diminished and regulated contact via living arrangements, potentially threatening adolescents’ need to be a causal agent in one’s own life, as well as their need for belonging within the family. Theoretically, it is expected that parents can provide a supportive environment in this regard, both individually (e.g., Soenens et al., 2017) and as one parental system (Beckmeyer et al., 2019; Rejaän et al., 2021b). To capture how parents affect adolescents’ divorce-specific experiences of autonomy and belonging over time, a focus on processes that occur within families is required. Currently, however, there is a lack of studies that disentangle such within-family effects from interfamilial differences. Therefore, the objective of this study was to further our understanding of how family dynamics relate to important developmental experiences for adolescents growing up in divorced families, thereby distinguishing between-family associations from within-family effects. Random-intercept cross-lagged panel models were used to test the effects of parents’ behaviors on adolescents’ sense of divorce-specific autonomy and belonging, including parenting behaviors in both parent-child subsystems and the interparental subsystem (Cox & Paley, 2003), and partitioning between-family differences from within-family effects (Hamaker et al., 2015).

Overall, the between-family associations mainly showed that the family is an important context for adolescent functioning post-divorce, where some adolescents are protected from the risk of maladjustment to a greater extent than others, based on whether mothers and fathers generally engage in warm and supportive parenting, and have formed a coparental relationship characterized by cooperation and low conflict. Regarding adolescents’ experiences of autonomy, it is important that parents are able to work together after their separation, as coparenting dynamics were stronger correlates than parents’ child-directed autonomy supportive behavior. Yet, to experience family belongingness, it is really the relationships with parents within households that count, independent of whether parents cooperate or refrain from conflict. Further, the general absence of within-family associations between (co)parenting and autonomy or belonging revealed that parents and adolescents in this study, after spending an average of about 6 to 7 years as a divorced family, did not mutually influence each other’s behavior or experiences over the course of this 18 month-study.

### Post-Divorce (Co)Parenting and Adolescent Autonomy and Belonging

#### Autonomy in decision-making on living arrangements

Across families, adolescents who experienced a stronger sense of autonomy in decisions on living arrangements typically had parents who indicated being more tolerant of their children having different opinions and ideas, and who managed to interact more cooperatively and harmoniously towards their coparent. Whereas these findings regarding the role of parental autonomy support are clearly in line with theoretical expectations – it is the parenting dimension that is most unique en most central to SDT (Ryan & Deci, 2000) – the findings of this study add to existing literature by also demonstrating the importance of *coparenting* dynamics for adolescent autonomy post-divorce. It is considered beneficial for youth when both parents after divorce are positively involved in their children’s lives within the context of cooperative and harmonious coparental relationships (Cox & Paley, 2003; Minuchin, 1974), as for instance has been found in relation to adolescent adjustment (Amato et al., 2010; Rejaän et al., 2021b). This study suggests this applies to adolescents’ divorce-specific sense of autonomy as well, where the collaboration between parents appears a prerequisite for involving adolescents in decisions, or where conflicts between parents hinder adolescents’ sense of agency and influence in family decisions, as was suggested based on qualitative research (see Berman, 2018, for a review).

#### Family belonging

Although coparenting dynamics were also expected to be associated with adolescents’ experiences of belonging, results showed that parenting dynamics were more important: Adolescents with a stronger sense of belonging generally had parents who displayed more warmth and were more autonomy supportive. These findings are in line with theoretical notions of how the combination of warmth and autonomy support can give rise to the harmonious satisfaction of needs (Soenens et al., 2017). Additionally, they correspond with prior research that suggested the quality and closeness of parent-adolescent relationships to be the most important predictors of adolescents’ family belonging (King & Boyd, 2016; King et al., 2018). After all, belongingness pertains to how adolescents feel accepted and understood in their parental household(s) (Leake, 2007). Even though coparental cooperation and lack of conflict may not contribute to adolescents’ belonging to maternal and paternal households in the most direct sense, these factors have been shown to strengthen parents’ abilities to shape their parenthood within their own households (e.g., Adamsons & Pasley, 2006; Feinberg, 2003), suggesting they could have indirect effects on adolescents’ belonging as well. However, in the current study, associations between coparenting and parenting were weak and only (inconsistently) significant for interparental conflict. Our findings indicated that regardless of whether the coparental relationship is characterized by cooperation or conflict, adolescents can experience family belonging.

### A Matter of Stable between-Family Differences

Contrary to expectations (i.e., Ryan & Deci, 2000), the current study did not provide evidence for any longitudinal effects of (co)parental thwarts or supports on adolescents’ divorce-specific experiences of autonomy or belonging. Put differently, the links between (co)parenting and adolescent autonomy and belonging appeared to be a matter of stable differences between participating families, not of within-family processes in which adolescents and parent(s) prompted fluctuations in each other’s experiences or behaviors. One exception is the association between parental warmth and adolescents’ belonging, which changes were correlated. The lack of within-family effects of (co)parenting on adolescents’ autonomy might be explained by the divorce-specific focus on autonomy in this study. Based on prior qualitative evidence (e.g. Berman, 2018), living arrangements were assumed to be a regularly recurring theme in divorced families with youth living at home, but this may not have been the case in this sample, therefore reducing the chances of finding within-family effects of (co)parenting. Alternatively, families deal with these issues in different yet stable ways, with certain families being more positive, having open communication, and supporting the autonomy of their children, and its members feeling a greater sense of belonging, with other families having less open communication, and showing less autonomy support. Thus, fluctuations within families in these behaviors might not immediately affect autonomy and belonging, and prior studies that failed to disentangle between-level from within-level effects may have overstated the existence of such effects (Boele et al., 2019; Vrolijk et al., 2020).

Still, the general absence of within-family effects in this study does not rule out that such effects were present prior to families’ participation in this research or shortly after the divorce (Vrolijk et al., 2020; Van Lissa & Keizer, 2020). It only showed that such change did not occur in these families in their natural setting over the course of this study. The covariates in our models provided some insights into certain developmental trends. In line with developmental expectations (Deci & Vansteenkiste, 2004), parents tended to display more warmth and cooperation when their children were younger, while younger adolescents tended to experience more belonging and less autonomy. Also, the more time had passed since the divorce, conflicts between parents had generally decreased, while parental warmth increased. Thus, it could be that throughout the history of parental and coparental interactions, stable interaction patterns have been formed. This particularly seems to apply to coparental dynamics, where there was little within-family variance, perhaps because in many families the divorce had taken place years ago. Prior research indeed showed that the reorganization of the coparental relationship typically takes time, but eventually becomes more stable (Fischer et al., 2005; Jamison et al., 2014). While our findings indicate that some families have been able to develop a good working mode, it also means that other families have developed rather rigid and problematic interaction patterns, which may be difficult to tackle.

### Associations between Autonomy and Belonging

Adolescents’ autonomy and belonging were also hypothesized to be closely interrelated, both for youth in general (Deci & Vansteenkiste, 2004), as well as specifically when growing up in a divorced family. When adolescents experience volition with respect to their living situation, this can help them feel included and understood within the family (Birnbaum & Saini, 2012; Lodge & Alexander, 2010). Also, in a supportive environment, adolescents may be more comfortable with expressing their views on current or desired living arrangements (Berman, 2018; Neale & Flowerdew, 2007). Indeed, findings showed that adolescents who on average experienced more autonomy regarding their living arrangements also tended to report a stronger sense of family belonging. Additionally, even though findings were not consistent, two out of four models indicated that individual fluctuations in adolescents’ autonomy and belonging were related within measurement waves. This means that when adolescents reported relatively more autonomy than they typically experienced, they also reported relatively more belonging at that time.

### Limitations and Directions for Future Research

Naturally, this study has several limitations that have to be taken into account when interpreting its findings. Firstly, the fact that father-reports were only available for a third of the participating adolescents at T1 resulted in the merging of father and mother data into single scores. The under-sampling of fathers is a common problem in (divorce) research, and more studies into the role of fathers’ parenting is needed. Ideally, adolescent outcomes are tested in relation to fathers’ and mothers’ (co)parenting separately, since it is known that there can be substantial variations in their parenting and coparenting behaviors that are not taken into account in the current study (e.g., Russell et al., 2016; Vrolijk et al., 2020). Furthermore, even though due consideration was given to the use of their self-reports, the use of cross-reports would have resulted in insights into potential discrepancies in parents’ and adolescents’ perceptions of (co)parenting (see Korelitz & Garber, 2016). Regarding the measurement of adolescents’ autonomy in terms of their living situation, a limitation was that items were only presented to adolescents who indicated that there were established living arrangements in their divorced families. This means that our findings are not applicable to adolescents from families where there are no formal or explicit agreements about the arrangement of care and contact, while also for these adolescents, a certain sense of autonomy in living arrangements is relevant and desirable.

In addition to the methodological directions for future research that can be derived from the limitations of this study, the results particularly raise the question of what family- or adolescent-factors can strengthen their sense of autonomy and belonging when growing up in a divorced or separated family. Perhaps even more specific measures are needed for disentangling within-family effects from between-family differences, for example by tailoring questions with regard to parental belongingness support (e.g., respect and warmth), competence support (e.g., offering clear expectations and adequate help), and autonomy support (e.g., providing choice and encouraging exploration) to divorce-specific situations, or studying links between (co)parenting and adolescents’ experiences of autonomy and belonging during transitional periods. Alternatively, an innovative perspective has recently been introduced: the notion of need crafting (Laporte et al., 2021). Rather than being solely dependent on supportive environments, need crafting entails awareness of one’s personal resources for support as well as a tendency to act upon this awareness, for instance by reflecting on who you are as a person and what is important to you, and committing to act upon these reflections. The first psychometric results are certainly promising for studying adolescents’ needs, as they show significant associations with (mal)adjustment at both the between- and within-person level, indicating that need crafting is susceptible to change, and therefore potentially valuable for intervention or prevention (Laporte et al., 2021).

## Conclusion

While there are numerous studies on parents’ role in the fulfillment of adolescents’ needs for autonomy and belonging, there are two major gaps: Both research on the dynamics that occur in divorced families and research on within-family processes are strikingly limited. The current study addressed these gaps by using random intercept cross-lagged panel models to examine whether post-divorce (co)parenting behaviors were longitudinally associated with adolescents’ perceptions of autonomy in decision-making on post-divorce living arrangements and their sense of belonging to the family household(s) in which they reside. Findings mainly showed differences between families, indicating that adolescents growing up in divorced families with less parental warmth and autonomy support, lower coparental cooperation and more conflict seem to be at higher risk for the thwarting of adolescents’ needs. An important contribution to existing literature is the finding that adolescents’ autonomy seems better safeguarded in divorced families where parents have established a more cooperative relationship, whereas parents seem individually tasked with supporting adolescents’ sense of belonging to their households. Two implications for practice can be derived from this. Firstly, parents and any professionals guiding them should be aware of the role parents play both through their parenting and coparenting in supporting their child’s need for autonomy following a divorce. Secondly, divorce programs should prioritize teaching parents individual parenting skills so that they can provide their children with a warm and supportive home environment, rather than focusing only on the coparental relationship after divorce. However, the second key finding is that hardly no significant associations of (co)parenting with adolescent autonomy and belonging emerged at the within-family level. Thus, when parents displayed more warmth or autonomy support towards their children and more cooperation and less conflict towards their ex-partner than usual, adolescents’ did not experience an increase in their sense of autonomy or belonging. Conversely, and perhaps more importantly for adolescents’ functioning in post-divorce families, contrary to theoretical expectations, the findings of this study indicate that temporary declines in the quality of (co)parenting are not necessarily harmful for the development of adolescents growing up in divorced families. What seems to matter most is the nurturing role of parents throughout childhood and adolescence: While some adolescents are lucky to have been able to grow up in a supportive home environment, others may require specialized care to strengthen (co)parental skills and family resources after a parental divorce.
